# An Age-Specific Crisis Awareness Survey of COVID-19 Vaccines and Treatments Among Healthy Japanese Adults

**DOI:** 10.7759/cureus.65549

**Published:** 2024-07-27

**Authors:** Takashi Kawahara, Teppei Takeshima, Kazuhide Makiyama, Hiroji Uemura

**Affiliations:** 1 Urology, Yokohama City University Medical Center, Yokohama, JPN; 2 Urology, Yokohama City University, Yokohama, JPN

**Keywords:** pandemic, covid-19, awareness, age, generation

## Abstract

Introduction and objectives

The spread of coronavirus disease 2019 (COVID-19) has been a worldwide phenomenon. This study conducted a chronological survey of crisis awareness regarding COVID-19 within different age groups.

Methods

An internet-based survey was performed on healthy Japanese adults to investigate the value of a hypothetical prophylactic vaccine and therapeutic drug for COVID-19 in each age group. In total, 12 groups comprising males and females in six different age groups, leading to a total of 1,200 individuals, were surveyed on a weekly basis. The survey was conducted on Friday of each week commencing from February 14, 2020, to April 10, 2020. At certain times or events such as when the government released major announcements or when there was a rapid increase in the number of infected individuals, a similar survey was conducted on an additional 1,200 individuals per week.

Results

A total of 12 surveys, including weekly surveys spanning over nine weeks, were conducted; a total of 19,113 samples from 12,254 individuals were obtained. The mean price for a hypothetical prophylactic vaccine was 2876.3 JPY (26.9 USD) at the first survey and was significantly increased to 3357.4 JPY (31.4 USD) for the most recent survey (p<0.0001). The percentage of healthy individuals who were unwilling to pay for a hypothetical therapeutic drug at the onset of COVID-19 symptoms was higher in the young age group than in the elderly groups at all phases, indicating a low level of crisis awareness among some young individuals. On the other hand, the percentage of those willing to pay for more than the standard prophylaxis influenza vaccine remained almost the same in all age groups, and it also increased when COVID-19 infection was widespread. In the sub-analysis, females who have children and married individuals tended to answer higher costs for prevention.

Conclusions

Changes in crisis awareness in all age groups were found to be associated with an increasing familiarity along with an increase in the number of locally infected individuals. Though the percentage of those who will not pay was higher in the young age group than in the elderly age group, the percentage of those who will pay more than the standard costs of influenza vaccine or treatment drugs was the same between each aging group.

## Introduction

The novel coronavirus infection, coronavirus disease 2019 (COVID-19) which first occurred in the Wuhan Province of China in 2019, has been spreading throughout the world since approximately February 2020 [1]. Although every country is trying to prevent the spread of COVID-19 through various isolation policies, the number of deaths continues to rise. As of April 16, 2020, the cumulative number of COVID-19 patients worldwide was 2,063,161, along with 136,938 deaths confirmed [2]. The risk of death is particularly high in individuals with underlying diseases and the elderly, while younger individuals often exhibit mild or asymptomatic disease with overall low mortality rates. Thus, the differences in crisis awareness in relevance to the disease between age groups have been an issue [3]. We therefore conducted a prospective study, starting on February 14, 2020, when only a small number of COVID-19 cases were noted in Japan, to determine how age range and the spread of infection affected crisis awareness.

This article was previously presented as a preprint in Research Square, posted on August 28, 2020. It was presented as a preprint in medRxiv as well, posted on April 27, 2020; however, the article has been removed from the medRxiv server now.

## Materials and methods

Screening survey

From February 14, 2020, a questionnaire-type survey form was administered via iBRIDGE’s Freeasy, an internet survey firm (Tokyo, Japan: iBRIDGE Corporation). Healthy individuals were randomly selected each week from 4.5 million panels at Freeasy based on age and gender parameters. Two questionnaires were administered to a total of 1,200 individuals on a weekly basis (weeks one to four) who were split into 12 groups comprising males and females in six different age groups (i.e., 20s-60s years, 70 years or older) because it was initially expected that the spread of COVID-19 would end early. Subsequently, a total of 1,200 individuals were administered weekly as it was expected that there would be a global spread and prolongation to the end of the spread of infections. In addition, 1,200 additional individuals were administered whenever the World Health Organization (WHO) or the Japanese government issued announcements or there were major changes in infection status. Since the questionnaire was, for the most part, completed within one day, it was considered that there would be no overlap in the questionnaire period. In addition to sex and age, information on prefecture/city of residence, type of residential facility, annual household income, marriage status, and the presence or absence of children were obtained together. Monitoring and evaluation were consented to during the online survey at the time of answering the questionnaire, while the Institutional Review Board (IRB) of Yokohama City University Medical Center (Yokohama, Japan) approved this study (IRB no. B200300041).

Questionnaire

To investigate crisis awareness regarding COVID-19 in healthy Japanese adults, the values assigned to a hypothetical COVID-19 prophylactic vaccine and hypothetical COVID-19 treatment drugs were compared to the price paid for the domestic influenza vaccine and other influenza drugs in Japan. Japan has a universal insurance system that is different from that of most other countries. Under this system, almost all Japanese nationals have access to uniform treatment regardless of employment status or income. Conversely, since the proportion of out-of-pocket payments varies based on income and insurance type, there is a difference in the amount paid by an individual who receives the same treatment (about 70-90% of medical costs are covered by insurance). The influenza vaccine is administered annually to approximately 50% of the population or 60 million Japanese nationals, and the average out-of-pocket amount for each individual in Japan is about 3,600 JPY (33.6 USD). Moreover, crisis awareness regarding COVID-19 in healthy individuals was investigated by asking them what they thought the expected payment should be for both a hypothetical prophylactic vaccine and hypothetical therapeutic drugs specific to COVID-19, assuming out-of-pocket expenses, including medical care and drugs, would be around 3,000-6,000 JPY (28.0-56.1 USD) (although there would be a range in the cost of treatment drugs). A crisis awareness survey consisted of two simple questions, “How much would you pay for a vaccine against COVID-19?” and “How much would you pay for treatment for COVID-19?” (Table 1).

**Table 1:** Questionnaire (translated to English).

Q1
Currently, a new type of pneumonia (coronavirus) has become a hot topic, with an increase in the number of infected people in Japan.
For example, influenza vaccinations are currently available at a price of approximately 3,600 JPY per injection in Japan. If you can get a coronavirus vaccination in the same way as an influenza vaccination, how much do you think you should pay?
(a) If you do not want to pay, please enter 0 JPY.
(b) If you are willing to pay, please enter the amount (once a year).
(c) Please indicate in the same way as when getting the influenza vaccination.
(d) The upper limit is 100,000 yen. If it is more than this, please enter 100,000 yen.
Q2
Currently, a new type of pneumonia (coronavirus) has become a hot topic, with an increase in the number of infected people in Japan.
For example, the price of flu drugs is 3,000-6,000 JPY per treatment (actual treatment fees, including those for consultation and examination, may vary depending on the co-payment ratio of various insurances).
If a coronavirus treatment drug is available, just like flu drugs, how much do you think you should pay?
(a) If you do not want to pay, please enter 0 JPY.
(b) If you are willing to pay, please enter the amount.
(c) Please enter the price, compared to 3,000-6,000 JPY for flu drugs.
(d) Please answer as if you are being treated as an outpatient without being hospitalized, as with influenza treatment.

The following parameters were compared as indices of crisis awareness: the number of deaths in Japan and around the world, and the cost of a hypothetical prophylactic vaccine and treatment drugs. The proportion of individuals who were willing or unwilling to pay more than the price of the influenza vaccine and drugs was also evaluated. Additional surveys were conducted to determine the impact of major announcements from the Japanese government, WHO, or similar bodies, as well as the timing of such events.

Comparison with the number of patients

The numbers of infected individuals and patient deaths in Japan and worldwide were based on those reported by the Japanese Ministry of Health, Labour, and Welfare and data from the Johns Hopkins Coronavirus Resource Center, respectively [4].

Statistical analyses

The participants’ characteristics and scores were analyzed by the Mann-Whitney U test using the GraphPad Prism software program (La Jolla, CA: GraphPad Software). P-values <0.05 were considered to be statistically significant.

## Results

**Figure 1:**
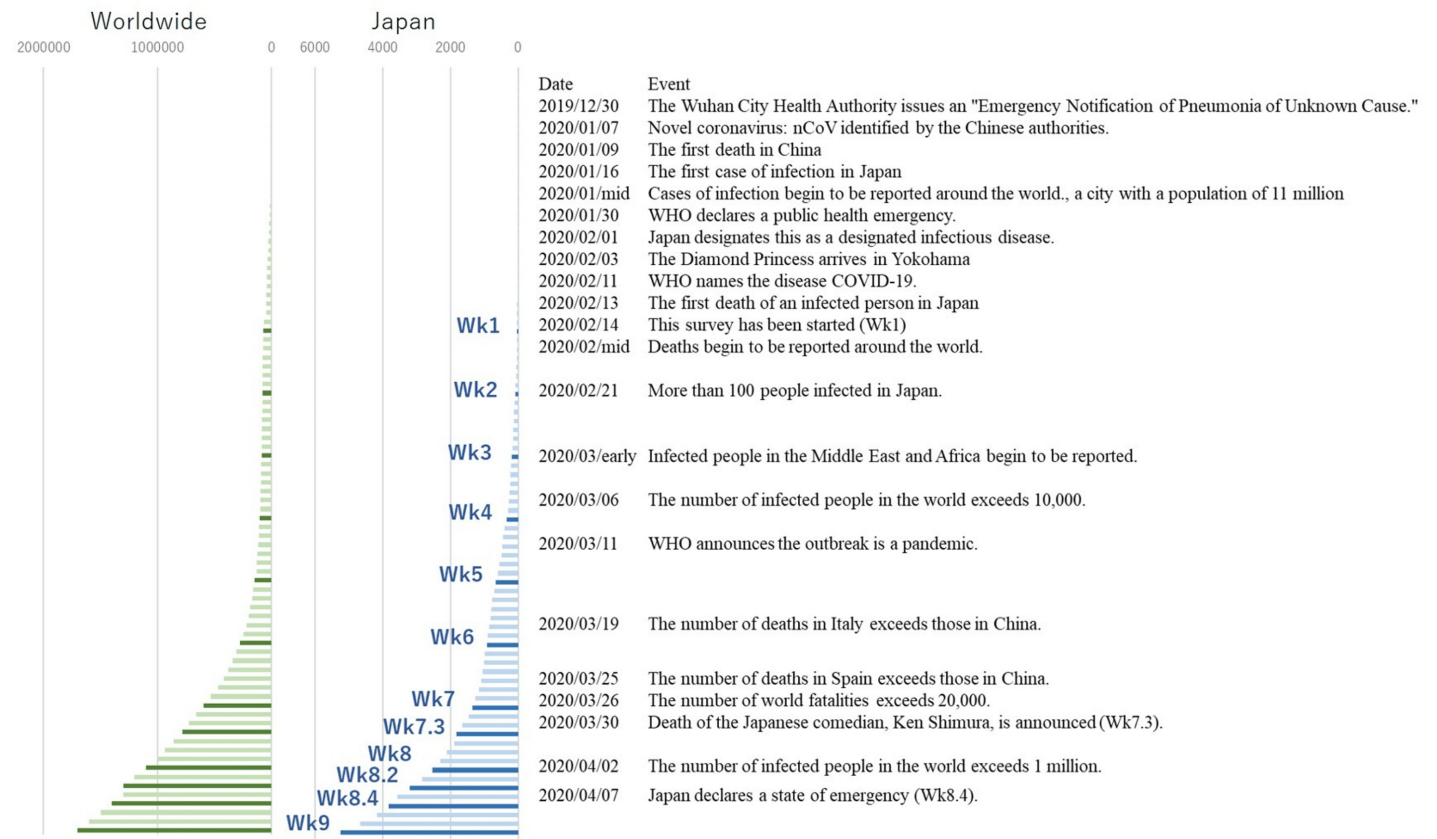
History of COVID-19 and number of patients (green: worldwide cases, blue: Japanese cases).

The study was conducted on 19,113 samples (12,254 individuals) with a total of 12 surveys in the timeframe between February 14, 2020, and April 12, 2020. These included weekly surveys that were administered every Friday, three additional surveys, and others administered during events such as major announcements from the government and when there was a drastic rise in the number of infected individuals (Figure 1, Table 2). On February 14, 2020 (week {Wk} 1), the number of individuals in Japan who were positive for COVID-19 test was only 33, and 63,851 (95.4%) out of 66,900 positive cases in the world were from China. On April 10, 2020 (Wk9), there was an explosive increase in the number of positive cases in Japan (5,246 individuals) and globally (1.7 million) (Figure 1).

**Table 2:** Background of the study samples.

Variables		Median (mean±SD) or number (%)
Number of samples (cases)		19,113 samples (12,254 cases)
Age (years)		49 (49.5±16.8)
Gender	Male	9,510 (49.8%)
	Female	9,603 (50.2%)
Married		11,428 (59.8%)
Child		9,732 (50.9%)
Household income (per year)	>2,000,000 JPY	3,177 (8.7%)
	2,000,001-4,000,000 JPY	5,235 (12.7%)
	4,000,001-6,000,000 JPY	4,494 (13.1%)
	6,000,001-8,000,000 JPY	2,613 (7.0%)
	8,000,001-10,000,000 JPY	2,391 (4.3%)
	10,000,001 JPY	1,203 (2.9%)
Working pattern	Housewife	3,556 (18.6%)
	Employee	5,277 (27.6%)
	Part-time job	2,471 (12.9%)
	Temporary job	982 (5.1%)
	No-occupation	3,560 (18.6%)
	Others	3,267 (17.1%)

**Figure 2:**
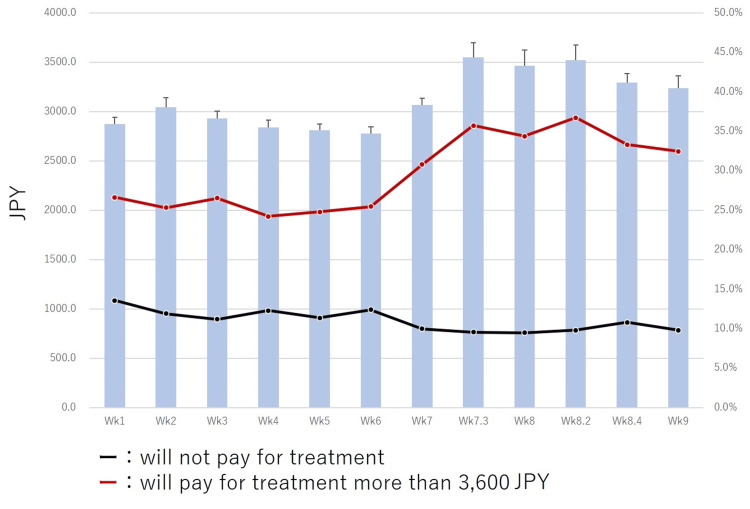
Transition of hypothetical COVID-19 vaccine cost (will pay for vaccination in JPY). Percentages of cases who would pay more than 3,600 JPY (red line) and who would not pay (black line) for this vaccine are shown. Over time, the proportion of those unwilling to pay for COVID-19 vaccine decreased; the proportion of those willing to pay more than the average cost of influenza vaccination for COVID-19 vaccine trended upward over time.

**Figure 3:**
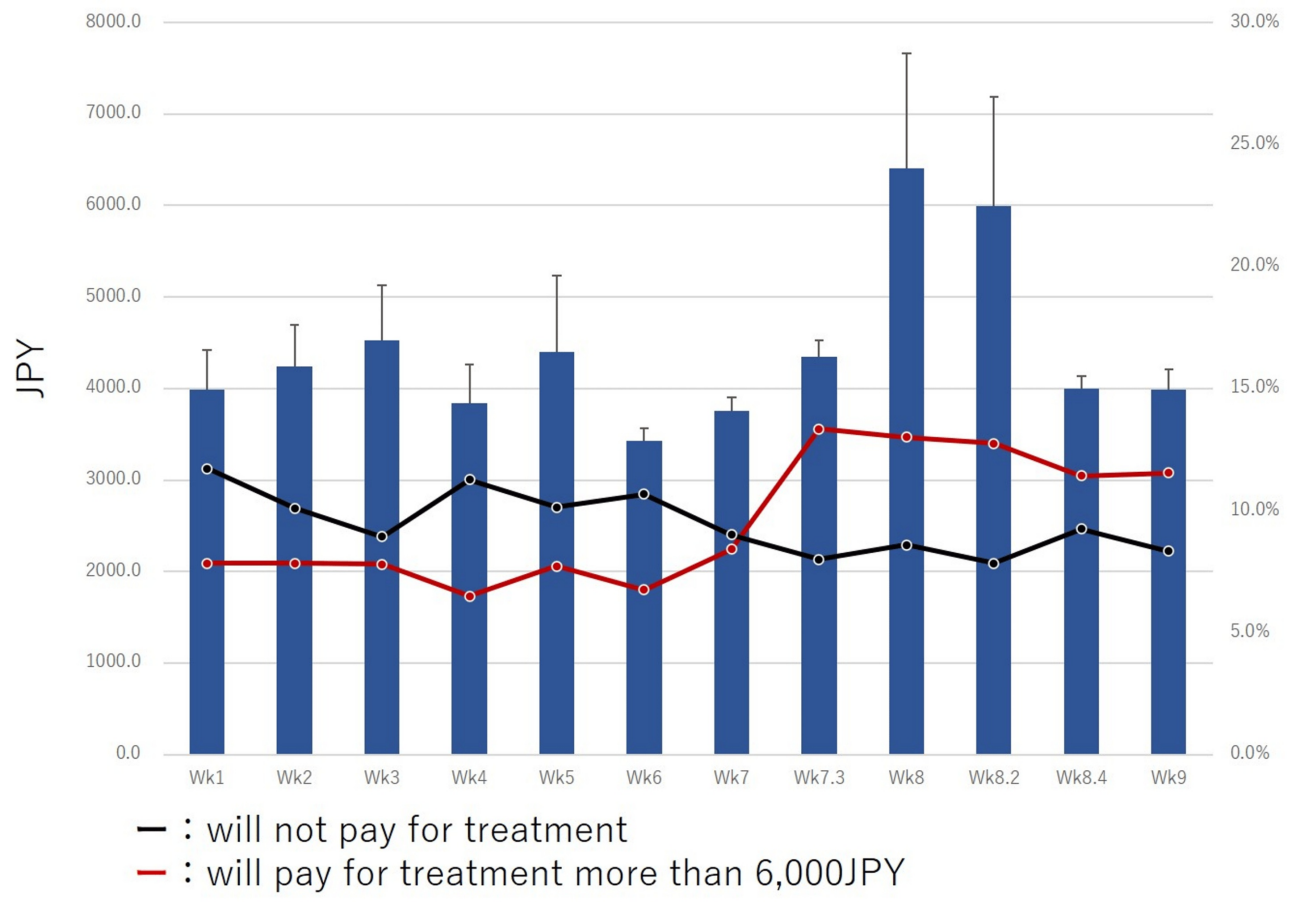
Transition of hypothetical COVID-19 treatment cost (will pay for treatment in JPY). Percentages of cases who would pay more than 6,000 JPY (red line) and who would not pay (black line) for this treatment are shown. Over time, the percentage of those unwilling to pay for COVID-19 treatment decreased. The percentage of those willing to pay more than 6,000 JPY for COVID-19 treatment increased over time.

In this study, healthy individuals answered the amounts they would consider paying for a hypothetical prophylactic vaccine and therapeutic drugs for COVID-19. Results showed that, as the number of COVID-19 infections increased, the prices allocated for the hypothetical vaccine and drugs increased till Wk8.2 (Figures 2, 3). However, when the Japanese government declared a state of emergency at Wk8.4, the prices allocated for the hypothetical vaccine and therapeutic drugs were decreased (Figures 2, 3). Prices for the hypothetical prophylactic vaccine for COVID-19 significantly increased from 2876.3 JPY (26.9 USD) at Wk1 to 3357.4 JPY (31.4 USD) at WK9 (p<0.001). A temporary decrease in the hypothetical prices was noted on March 20-22, 2020 (Wk6) during a long weekend when an increase in the number of infected individuals was reported in Japan. An increase in the hypothetical price was also seen in the report of the death of a famous Japanese comedian due to COVID-19 on March 30, 2020, at Wk7.3 (Figure 2).

**Figure 4:**
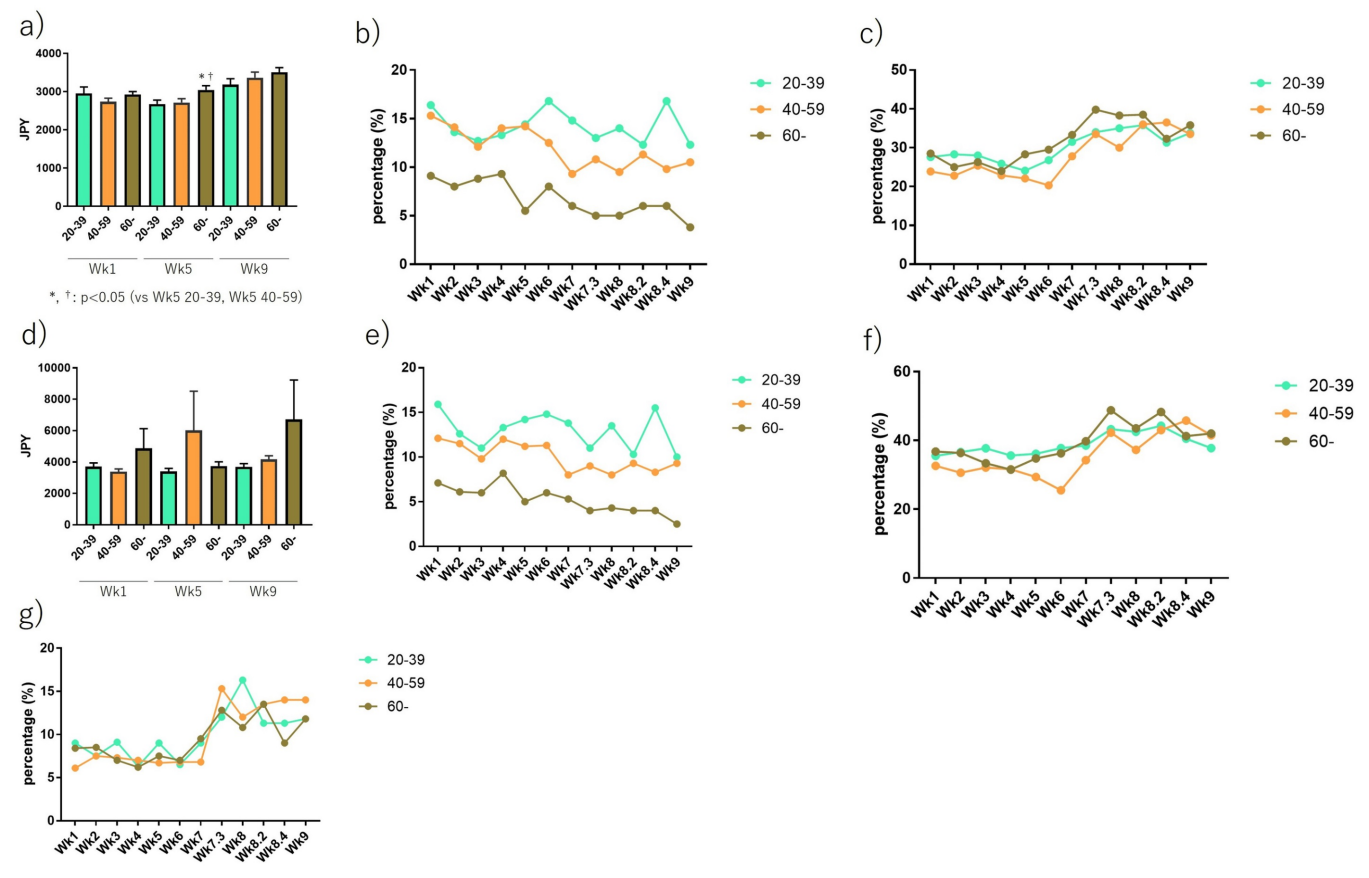
Comparison of COVID-19 prevention and treatment variations across different age groups. *p<0.05 vs 20-30. †p<0.05 vs 40-50. (a) Hypothetical prevention cost at Wk1, Wk5, and Wk9. In Wk5, 60 or more groups showed significantly higher values than both in 20-39 (p<0.05) and in 40-59 (p<0.05). (b) Percentage of cases who were not willing to pay for a vaccine. (c) Percentage of cases who were willing to pay more than 3,600 JPY for a vaccine. (d) Hypothetical treatment cost at Wk1, Wk5, and Wk9. (e) Percentage of cases who were not willing to pay for the treatment. (f) Percentage of cases who were willing to pay more than 3,600 JPY for the treatment. (g) Percentage of cases who were willing to pay more than 6,000 JPY for the treatment.

Across the different age groups, the prices for the hypothetical prophylactic vaccine for COVID-19 have been significantly higher in the elderly group than in the younger age group at Wk5 when the number of infected individuals domestically exceeded compared to the previous several weeks (Figure 4a). The percentage of those who were not willing to pay for prevention was higher in the young group compared to the elderly group, but the percentage of those who were willing to pay for prevention more than the prophylaxis influenza vaccine was almost the same between each age group. When the number of positive COVID-19 cases increased in Japan, the percentage of those who were willing or unwilling to pay for prevention increased or decreased, respectively (Figures 4b, 4c). For the treatment, the elderly group was willing to pay more but there were no significant differences (Figure 4d). The percentages of those who were not willing to pay for prevention were higher in the young group and lower in the elderly group. The percentage of all participants who were not willing to pay for the treatment decreased when COVID-19 was widespread (Figure 4e). The percentage of those who were willing to pay for the treatment was similar in all age groups during all phases but gradually increased as COVID-19 was widespread (Figures 4f, 4g).

**Figure 5:**
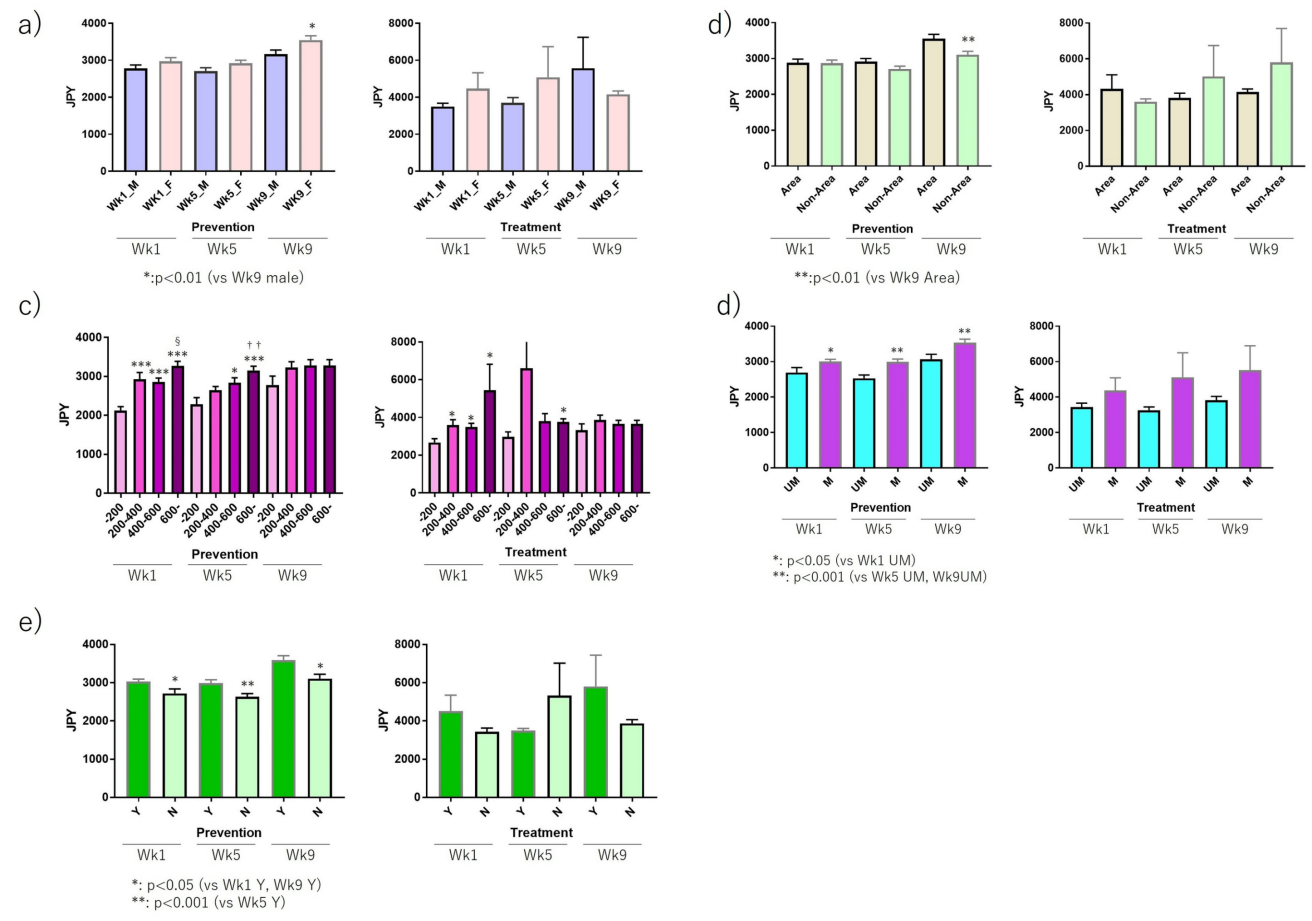
The differences in the willingness to pay for prevention and treatment of COVID-19. §p<0.05 vs 200-400. ††p<0.01 vs 400-600. (a) Gender, (b) area, (c) annual household income, (d) marriage status, and (e) presence of child(ren). In Wk9, female individuals would pay more value than males (p<0.01) for prevention. (b) In Wk9, the non-area group showed that they would pay less than the area group (p<0.01) for prevention. (c) In Wk1 and Wk5, the household income higher group would pay more than the household income lower group (200-400 JYP, p<0.001, p<0.001; 400-600 JYP, p<0.001, p<0.001; >600 JYP, p<0.001, p<0.001 vs <200 JYP group) for prevention. In Wk1 and Wk5, the household income higher group would pay more than the household income lower group (200-400 JYP, p<0.05, p<0.05; 400-600, p<0.05, p<0.05; >600 JYP, p<0.05, p<0.05 vs <200 JYP group) for treatment. (d) In Wk1, Wk5, and Wk9, married individuals would pay more than in the unmarried group (p<0.05, p<0.001, p<0.001) for prevention. (e) In Wk1, Wk5, and Wk9, the non-presence of child group would pay less than in the individual with child (p<0.05, p<0.001, p<0.05) for prevention.

In further subgroup analysis, the percentage of females willing to pay more than the standard influenza vaccine for prevention was significantly higher at Wk9 compared to males (Figure 5a). At Wk8.4, the Japanese government declared a state of emergency in seven prefectures. When compared between these seven prefectures (area) and the other area (non-area), there were no differences before the state of emergency, but after the state of emergency, those in the area were willing to pay more than those in the non-area (Figure 5b). In terms of household income, in an early era, higher household income groups were willing to pay higher amounts, but the differences were then diminished (Figure 5c). In addition, those who were married and had a child/children were more likely willing to pay for prevention at all phases (Figures 5d, 5e).

## Discussion

This study was a large-scale survey of 19,113 samples and 12,254 individuals over nine weeks. Although low levels of crisis awareness in younger age groups have been reported in media coverage and other small-scale awareness surveys, this was the first objective prospective survey to investigate this topic. We found that the increasing spread of COVID-19 throughout Japan more strongly correlated with an increased potential awareness of prevention and treatment for COVID-19 than the spread of COVID-19 worldwide. Additionally, a deeper awareness of treatment further increased with the reported death of a famous Japanese celebrity comedian on March 30 (Wk7.3). Reports on the rise of individuals who tested positive for COVID-19 from the Diamond Princess cruise line from February 5 to March 1 (Wk1-3) temporarily resulted in increased crisis awareness. In these periods, there were no significant differences in crisis awareness, and the cost of hypothetical vaccines and drugs showed an almost flat trend in not only the younger age group but also the elderly.

In Japan, as there was no significant rise in the number of infected individuals in these survey times, there seemed to be a sense of security in individuals. These more individuals, combined with the fact that it was also cherry blossom viewing season, ventured outside during the long weekend from March 20-22. In this study, the prices allocated for the hypothetical prophylactic vaccine and other therapeutic drugs were also reduced on March 20 (Wk6), and the survey seemed capable of reflecting crisis awareness in Japanese individuals. Moreover, an additional survey conducted at the time of the reported death of a famous comedian in Japan showed a sharp rise in awareness.

Differences in crisis awareness between the younger age group and the elderly were believed to result from age-related differences, such as the increase in severity of symptoms and higher mortality rates related to age. Mortality rates associated with COVID-19 increased with age, with 0.2% deaths in those in their 20s or 30s, 0.4% in those in their 40s, 1.3% in those in their 50s, 3.6% in those in their 60s, 8.0% in those in their 70s, and 14.8% in those 80 years or older [5]. For this reason, the assumption is that the younger age group is less aware of the crisis and consequently has lower compliance rates to governmental directives, such as bans on going outside. However, the percentage of cases who were not willing to pay for the prevention and treatment was reduced correlating with increases in the number of COVID-19 positivity in Japan. The proportions of participants who were willing to pay for the treatment more than the standard price for the influenza drugs in any phase were similar in all age groups and increased with widespread COVID-19.

In this study, crisis awareness was evaluated based on a comparison of the cost of prophylactic and treatments with that for influenza. There are currently no questionnaires that assess awareness of treatment and sense of crisis that are validated in the Japanese language. It is currently unknown whether questionnaires would be suitable for this infectious disease. In this regard, the idea was that performing a comparison to those of influenza, an infectious disease for which prophylactic vaccination and treatment were provided in about 50% of individuals in Japan would be convenient. Japan ranks 16th in the Organization for Economic Co-operation and Development members in influenza vaccination rate in those aged 65 years, with the Republic of Korea ranking highest at 83%, followed by Australia, the United Kingdom, and the United States [6,7]. Specifically, in 2020, the percentage of people vaccinated for influenza was 58.5% in the elderly, 28.6% in adults, and 59.2% in children [6,7]. It is also considered easy to assess the number of infected individuals since there has been a constant frequency of 10 million individuals in Japan each year [8]. Thus, we used this influenza comparing questionnaire due to the popular disease in Japan. Conversely, the mean cost of a hypothetical prophylactic vaccine and other hypothetical prophylactic drugs were 2876.3 JPY (26.9 USD) and 3357.4 JPY (31.4 USD), respectively, at Wk9 and considerably lower in situations where the government declared an emergency compared to seasonal influenza.

This study evaluated the demand for medical care in healthy individuals. In particular, in patients with severe symptoms of COVID-19, the medical load is higher with measures in place such as ventilator management and this higher load can lead to a higher burden on medical workers. Consequently, it may also be the cause of medical disruption which can lead to higher mortality rates. Since Japan, in particular, still experiences lower morbidity and mortality compared to Western countries at the time of this study, there is a belief that raising awareness among individuals without symptoms will be necessary.

The COVID-19 pandemic had a major impact not only on deaths among infected individuals but also among non-infected individuals. This includes not only physical and morbidity but also economic impact, including prevention of infection [9-11]. This study also examines the costs associated with infection prevention due to various social contexts, which we believe will provide valuable experience on how to allocate prophylaxis in the event of future outbreaks following COVID-19.

Limitations

The validity of the questionnaire itself and the lack of detailed evaluation including the ability to perform evaluations over time despite issues with awareness remaining are some of the limitations of this study. However, this simple survey could evaluate the results regardless of final educational attainment.

The study revealed a change in crisis awareness in all age groups, which was associated with an increasing awareness of infected individuals. Moreover, crisis awareness was lower in the younger age group than in the elderly, whereas crisis awareness in the middle-aged group fluctuated between high and low levels. Meanwhile, females, married persons, and those with child(ren) showed higher crisis awareness.

## Conclusions

Changes in crisis awareness in all age groups were found to be associated with an increasing familiarity along with an increase in the number of locally infected individuals. Though the percentage of those who will not pay was higher in the young age group than in the elderly age group, the percentage of those who will pay more than the standard costs of influenza vaccine or treatment drugs was the same between each aging group.
